# Understanding the Relationship Between Nomophobia and Psychological Distress Among University Students: The Sequential Mediating Roles of Social Anxiety and Loneliness

**DOI:** 10.3390/healthcare14162441

**Published:** 2026-08-07

**Authors:** Bandar S. Alharbi, Majed M. Aljabri, Alya Alghamdi

**Affiliations:** Community and Psychiatric Mental Health Nursing Department, College of Nursing, King Saud University, Riyadh 12375, Saudi Arabia; amajad@ksu.edu.sa (M.M.A.); aalgamdii@ksu.edu.sa (A.A.)

**Keywords:** nomophobia, social anxiety, loneliness, psychological distress, structural equation modeling, university students, Saudi Arabia

## Abstract

**Background**: Nomophobia, the fear or anxiety associated with being unable to access or use a smartphone, has become a serious public health concern among university students. Although previous studies have reported associations between nomophobia and adverse mental health outcomes, the psychological mechanisms underlying these relationships remain poorly understood. This study examined the associations among nomophobia, social anxiety, loneliness, and psychological distress and evaluated the sequential mediating roles of social anxiety and loneliness. **Methods**: We used a cross-sectional study among 254 university students enrolled in the three public universities in Riyadh, Saudi Arabia. Participants completed an online questionnaire assessing nomophobia, social anxiety, loneliness, and psychological distress using validated instruments. Structural equation modeling was performed to estimate the direct and indirect associations among the latent constructs. Model fit was assessed using the Comparative Fit Index (CFI), Tucker–Lewis Index (TLI), Root Mean Square Error of Approximation (RMSEA), and Standardized Root Mean Square Residual (SRMR). **Results**: The integrated measurement model showed excellent fit to the data (χ^2^ = 326.57, df = 179, χ^2^/df = 1.82, CFI = 0.993, TLI = 0.992, RMSEA = 0.057, and SRMR = 0.053). The nomophobia dimension reflecting concerns related to information and communication access was positively associated with social anxiety (β = 0.228, *p* = 0.006), whereas the second nomophobia dimension was not significantly associated with social anxiety. Social anxiety was positively associated with loneliness (β = 0.561, *p* < 0.001), and loneliness was positively associated with psychological distress (β = 0.479, *p* < 0.001). The sequential indirect association between the first nomophobia dimension and psychological distress through social anxiety and loneliness was statistically significant (β = 0.067, *p* = 0.042). No significant sequential indirect association was observed for the second nomophobia dimension. **Conclusions**: Among university students, concerns related to losing access to information and communication were associated with greater psychological distress indirectly through increased social anxiety and loneliness. Interventions aimed at improving students’ mental well-being may benefit from addressing both social anxiety and loneliness in addition to promoting healthy smartphone use. Longitudinal studies are warranted to confirm the temporal relationships among these constructs.

## 1. Introduction

The rapid increase in digital technology has fundamentally transformed the way people communicate, access information, learn, and interact with society [1]. Smartphones have become indispensable tools in everyday life, particularly among university students, who rely on them for academic activities, social networking, entertainment, financial transactions, and access to health information [2]. Although smartphones provide substantial educational and social benefits, excessive reliance on these devices has raised increasing concerns regarding their potential effects on psychological well-being. Several studies indicate that problematic smartphone use is associated with impaired academic performance, reduced sleep quality, decreased social functioning, and a wide range of mental health problems [2,3,4].

One of the most prominent psychological consequences of excessive smartphone dependence is nomophobia, defined as the fear, anxiety, or discomfort experienced when individuals are unable to use or access their mobile phones [5]. Originally conceptualized as a modern technological phobia, nomophobia extends beyond simple device dependence and reflects psychological distress arising from concerns about losing communication, access to information, social connectedness, and convenience. University students are particularly vulnerable because smartphones have become deeply integrated into their academic, professional, and social lives. Consequently, interruption of smartphone access may evoke anxiety, insecurity, and emotional discomfort that interfere with daily functioning [6].

Recent studies indicate that nomophobia is highly prevalent among university students worldwide, with particularly high estimates reported in Middle Eastern countries, including Saudi Arabia [7,8]. The widespread availability of smartphones, extensive internet coverage, and the increasing use of digital technologies for education following the COVID-19 pandemic have further intensified smartphone dependence among young adults. In Saudi Arabia, where smartphone ownership among university students is nearly universal, several studies have reported moderate to severe levels of nomophobia [8]. Despite increasing recognition of nomophobia in the region, much of the available literature has focused on estimating prevalence or identifying demographic correlates, with relatively limited attention given to the psychological mechanisms through which nomophobia may influence mental health outcomes.

Recent evidence from systematic reviews and meta-analyses indicates that nomophobia has become highly prevalent among university students worldwide. A meta-analysis of 28 studies involving more than 11,000 university students reported that approximately 56% experienced moderate nomophobia and 17% experienced severe nomophobia, highlighting the substantial global burden of problematic smartphone dependence in this population [9]. More recent evidence, including over 36,000 participants from 18 countries, similarly confirmed that approximately one in two individuals experience moderate nomophobia and one in five experience severe nomophobia, reinforcing the consistency of these findings across diverse settings [10].

The systematic reviews and meta-analyses have further demonstrated that nomophobia represents a substantial and growing public health concern among university students [9,10]. Evidence synthesized from large international studies indicates that moderate to severe nomophobia affects a considerable proportion of university students across diverse cultural settings. These reviews also consistently report positive associations between nomophobia and anxiety, depression, stress, poor sleep quality, and reduced psychological well-being.

Among the potential mechanisms linking nomophobia to mental health, social anxiety represents one of the most plausible pathways. Social anxiety is characterized by excessive fear of negative evaluation, embarrassment, or scrutiny in social situations, often resulting in avoidance of interpersonal interactions. Individuals experiencing greater social anxiety frequently prefer digital communication because it provides greater control over social interactions and reduces perceived interpersonal risks [11].

Another important pathway involves loneliness, a subjective feeling arising from a discrepancy between desired and actual social relationships. Despite providing continuous digital connectivity, smartphones do not necessarily promote meaningful interpersonal relationships. Excessive smartphone use may reduce the quality of face-to-face interactions, increase social comparison through social media, and contribute to feelings of emotional isolation [12]. Individuals with high nomophobia may become increasingly dependent on virtual communication while simultaneously experiencing reduced social connectedness in their offline lives [13].

Psychological distress, encompassing symptoms of anxiety and depression, represents one of the most important indicators of mental health among university students [14]. The transition to university life is accompanied by substantial academic, financial, and social challenges that place students at increased risk of emotional difficulties. Previous studies have consistently shown that loneliness and social anxiety are independently associated with elevated psychological distress [15,16]. However, relatively few investigations have examined how these constructs operate together within a unified theoretical framework. Specifically, it remains unclear whether social anxiety and loneliness represent sequential psychological processes through which nomophobia contributes to emotional distress. Understanding these psychological pathways within the Saudi university context may provide valuable evidence for developing targeted mental health promotion strategies and interventions designed to improve students’ psychological well-being.

Therefore, we aimed to examine the associations among nomophobia, social anxiety, loneliness, and psychological distress among university students enrolled in the three public universities in Riyadh, Saudi Arabia. Specifically, we evaluated whether social anxiety and loneliness sequentially mediated the association between nomophobia and psychological distress while adjusting for selected sociodemographic characteristics and smartphone use behaviors. By identifying the psychological pathways underlying these associations, this study seeks to provide evidence that may inform university-based mental health promotion programs and interventions targeting problematic smartphone dependence among young adults.

## 2. Methods

### 2.1. Study Design and Setting

We conducted a cross-sectional study design among university students enrolled in the three major public universities in Riyadh, Saudi Arabia. Data were collected through a self-administered online questionnaire distributed to eligible students across participating universities. The data collection period was from 13 May 2026 to 22 May 2026. Unlike studies conducted within a single institution, we recruited participants from all three public universities in Riyadh, thereby increasing the diversity of the study population with respect to academic disciplines, educational backgrounds, and student characteristics. Including students from multiple institutions enhances the representativeness of the sample within the Riyadh university population and improves the external validity of the findings.

### 2.2. Participants and Sampling

The target population included undergraduate and postgraduate students enrolled in the three public universities in Riyadh, Saudi Arabia, during the study period. Eligible participants were students aged 18 years or older who were currently registered at one of the participating universities, able to read and understand English, and willing to provide informed consent. Students who declined electronic informed consent or submitted incomplete questionnaires were excluded from the analysis.

A convenience sampling strategy was employed. An open, university-wide recruitment strategy was implemented across all three participating public universities. Quick Response (QR) codes linking directly to the online questionnaire were displayed on official student announcement boards located in academic buildings and common student areas throughout each university. Students who scanned the QR code were directed to the electronic participant information sheet, informed consent form, and questionnaire. This recruitment approach provided all eligible students attending the participating universities with the opportunity to voluntarily participate during the study period while ensuring anonymous participation. After scanning the QR code, potential participants were directed to an electronic informed consent page. Only those who voluntarily provided electronic informed consent were granted access to the questionnaire. Participants who did not provide consent could not proceed to the survey and were not included in the study.

### 2.3. Data Collection

The questionnaire consisted of five sections covering sociodemographic characteristics, smartphone use, nomophobia, social anxiety, loneliness, and psychological distress. Before accessing the questionnaire, participants were presented with an electronic participant information sheet describing the study objectives, voluntary participation, confidentiality, and data protection procedures. Electronic informed consent was obtained before the questionnaire could be completed. No personally identifiable information was collected.

Participants reported their age, gender, university, college, academic year, average daily smartphone use, and primary smartphone activities. Average daily smartphone use was categorized as ≤4 h, 4 to 8 h, and >8 h. Participants were also asked to indicate their common smartphone activities, including social media, messaging and calls, entertainment, educational activities, news and web browsing, and gaming. Multiple responses were permitted for smartphone activities.

To assess nomophobia, we used a validated tool called the Nomophobia Questionnaire (NMP-Q), a self-administered instrument originally developed by Çağlar Yıldırım and Ana-Paula Correia to measure the anxiety, discomfort, or distress experienced when individuals are unable to use or access their smartphones [17]. The original NMP-Q consists of 20 items organized into four conceptual domains: not being able to communicate, losing connectedness, not being able to access information, and giving up convenience. Each item is scored on a seven-point Likert scale ranging from 1 (“strongly disagree”) to 7 (“strongly agree”), with higher scores indicating greater levels of nomophobia. The original instrument has showed excellent psychometric properties, including high internal consistency and construct validity among university students.

The NMP-Q has been widely used to assess nomophobia among university students and young adults in Middle Eastern countries, including Saudi Arabia, where it has shown good reliability and construct validity in studies investigating smartphone dependence and its psychological correlates [18,19]. Although the original NMP-Q comprises 20 items across four conceptual domains, our study evaluated an abbreviated measurement model consisting of 11 items representing two broader latent dimensions: concerns related to losing access to information and communication (e.g., inability to access information, receive messages, or communicate with family and friends). This abbreviated structure was evaluated for use in the present structural equation model and should not be interpreted as a replacement for the original validated instrument. The retained items were selected to capture the conceptual domains examined in the hypothesized model, and their psychometric performance was subsequently evaluated through confirmatory factor analysis, internal consistency, composite reliability, and convergent validity.

Responses to the 11 items were summed to generate a total nomophobia score ranging from 11 to 77, with higher scores indicating greater nomophobia. For the structural equation modeling analyses, however, nomophobia was operationalized using the validated two-factor latent measurement model identified through confirmatory factor analysis, comprising the dimensions of Information and Communication Access and Continuous Connectedness. The psychometric performance of the retained items was subsequently evaluated using confirmatory factor analysis together with assessments of internal consistency, composite reliability, and convergent validity before inclusion in the structural equation model.

We used the Mini Social Phobia Inventory (Mini-SPIN), a brief self-report screening instrument developed by Jonathan R. T. Davidson, Kathleen M. Connor, and colleagues, to identify individuals with generalized social anxiety disorder [20]. The Mini-SPIN consists of three items assessing the principal cognitive and behavioral features of social anxiety: fear of embarrassment or appearing foolish in front of others, avoidance of situations in which one may become the center of attention, and fear of interacting with unfamiliar people. Each item is rated on a five-point Likert scale ranging from 1 (“not at all”) to 5 (“extremely”), yielding a total score ranging from 3 to 15. In this study, the Mini-SPIN was used to assess social anxiety symptoms among university students. Total scores were calculated by summing responses across the three items, with higher scores representing greater social anxiety.

Loneliness was assessed using the three-item version of the University of California, Los Angeles (UCLA) Loneliness Scale, developed by Mary E. Hughes and colleagues as a brief measure of perceived social isolation for use in large epidemiological and population-based studies [21]. The three-item instrument was derived from the original 20-item UCLA Loneliness Scale developed by Daniel Russell and colleagues and was designed to retain the conceptual integrity of the original instrument while substantially reducing respondent burden [22]. The scale measures the subjective experience of loneliness through three core dimensions: lacking companionship, feeling left out, and feeling isolated from others. Specifically, participants were asked how often they (1) lacked companionship, (2) felt left out, and (3) felt isolated from others. Each item is rated on a three-point Likert scale ranging from 1 (“hardly ever”), 2 (“some of the time”), to 3 (“often”), with a total score ranging from 3 to 9.

Psychological distress was measured using the four-item Patient Health Questionnaire (PHQ 4), developed by Kroenke and colleagues [23]. The PHQ 4 combines the two-item Generalized Anxiety Disorder scale (GAD 2) and the two-item Patient Health Questionnaire depression scale (PHQ 2) into a brief screening instrument for anxiety and depressive symptoms. Participants rated each item on a four point Likert scale ranging from 0 (not at all) to 3 (nearly every day). Total scores range from 0 to 12 in the original PHQ-4. Higher scores indicate greater psychological distress.

### 2.4. Statistical Analysis

Categorical variables were summarized as frequencies and percentages, whereas continuous variables were described using means and standard deviations (SDs). We also performed correlation.

The psychometric properties of the measurement instruments were evaluated before structural modeling. Internal consistency was assessed using ordinal Cronbach’s alpha and McDonald’s omega. Composite reliability (CR) and average variance extracted (AVE) were calculated to evaluate construct reliability and convergent validity, respectively. CR values ≥ 0.70 and AVE values ≥ 0.50 were considered indicative of acceptable construct reliability and convergent validity.

Integrated confirmatory factor analysis (CFA) was conducted to evaluate the measurement model comprising the two latent nomophobia dimensions together with the latent constructs of social anxiety, loneliness, and psychological distress. Because all measurement items were ordinal, the model was estimated using the diagonally weighted least squares estimator with robust standard errors and mean- and variance-adjusted chi-square statistics (WLSMV), which is recommended for ordinal categorical data. Standardized factor loadings were examined to evaluate indicator reliability, and all retained indicators were required to demonstrate statistically significant factor loadings.

The hypothesized association among the latent constructs was subsequently examined using structural equation modeling (SEM). The structural model evaluated both the direct and indirect associations between the two dimensions of nomophobia and psychological distress, while considering social anxiety and loneliness as sequential mediators. The model was adjusted for potential confounding variables, including gender, college, and average daily smartphone use. Direct, indirect, and sequential indirect effects were estimated simultaneously using standardized path coefficients (β), standard errors (SE), 95% confidence intervals (CI), and corresponding *p* values. Because the structural equation model was estimated using the WLSMV estimator, standard errors and confidence intervals for the indirect effects were computed using the delta method implemented in the *lavaan* package. Sequential mediation was evaluated by estimating the pathway from nomophobia to psychological distress through social anxiety followed by loneliness.

The adequacy of both the measurement and structural models was evaluated using multiple goodness of fit indices, including the chi square statistic (χ^2^), degrees of freedom (df), the ratio of χ^2^ to df (χ^2^/df), the Comparative Fit Index (CFI), Tucker–Lewis Index (TLI), Root Mean Square Error of Approximation (RMSEA), and the Standardized Root Mean Square Residual (SRMR). Model fit was considered acceptable when CFI and TLI were at least 0.90, RMSEA and SRMR were below 0.08, and χ^2^/df was less than 3.0. Standardized path coefficients were interpreted to quantify the strength and direction of the relationships among the latent constructs.

All statistical analyses were performed using R statistical software (version 4.4.3; R Foundation for Statistical Computing, Vienna, Austria) [24].

## 3. Results

A total of 254 university students, both male (55.1%) and female (44.9%), participated in the study (Table 1). Most participants were 18 to 23 years of age (70.9%), with the remaining 29.1% aged 24 years or older. The majority of the respondents were enrolled in Years 3 to 4 of their academic program (74.0%), whereas 19.7% were in Years 1 to 2 and 6.3% were in Year 5 or postgraduate studies. Nearly half of the participants were from the Health Sciences (45.3%), followed by Business (16.1%), Engineering/Science (13.8%), Education (13.8%), and Humanities/Other (11.0%). Most students reported using their smartphones for 4 to 8 h per day (65.4%), while 21.7% reported using their smartphones for more than 8 h daily. Only 13.0% reported using their smartphones for 4 h or less per day. Social media use was the predominant smartphone activity (95.3%), followed by messaging and calls (67.7%) and entertainment (65.7%). Educational use was reported by 40.9% of participants, while approximately one-quarter used their smartphones for news and browsing (26.4%) or gaming (24.4%).

The mean nomophobia score was 48.93 ± 12.94. The mean social anxiety score was 7.85 ± 2.40, the mean loneliness score was 5.80 ± 1.53, and the mean psychological distress (PHQ-4) score was 8.82 ± 2.38. On average, participants reported moderate levels of nomophobia, social anxiety, loneliness, and psychological distress.

The psychometric properties of the study instruments are given in Supplementary Table S1. The scales showed acceptable to excellent internal consistency and convergent validity. The two nomophobia dimensions exhibited excellent reliability, with ordinal Cronbach’s α values of 0.902 for the Information dimension and 0.918 for the Communication dimension. The corresponding McDonald’s ω values were 0.901 and 0.909. The Social Anxiety scale also demonstrated acceptable reliability, with an ordinal Cronbach’s α of 0.787 and McDonald’s ω of 0.748. Similarly, the Psychological Distress scale showed acceptable internal consistency (ordinal Cronbach’s α = 0.804; McDonald’s ω = 0.754). The Loneliness scale demonstrated acceptable reliability based on the ordinal Cronbach’s α (0.752). Composite reliability values ranged from 0.688 to 0.909. Average variance extracted (AVE) values ranged from 0.510 to 0.703, exceeding the recommended cutoff of 0.50 for all constructs and indicating adequate convergent validity.

**Figure 1:**
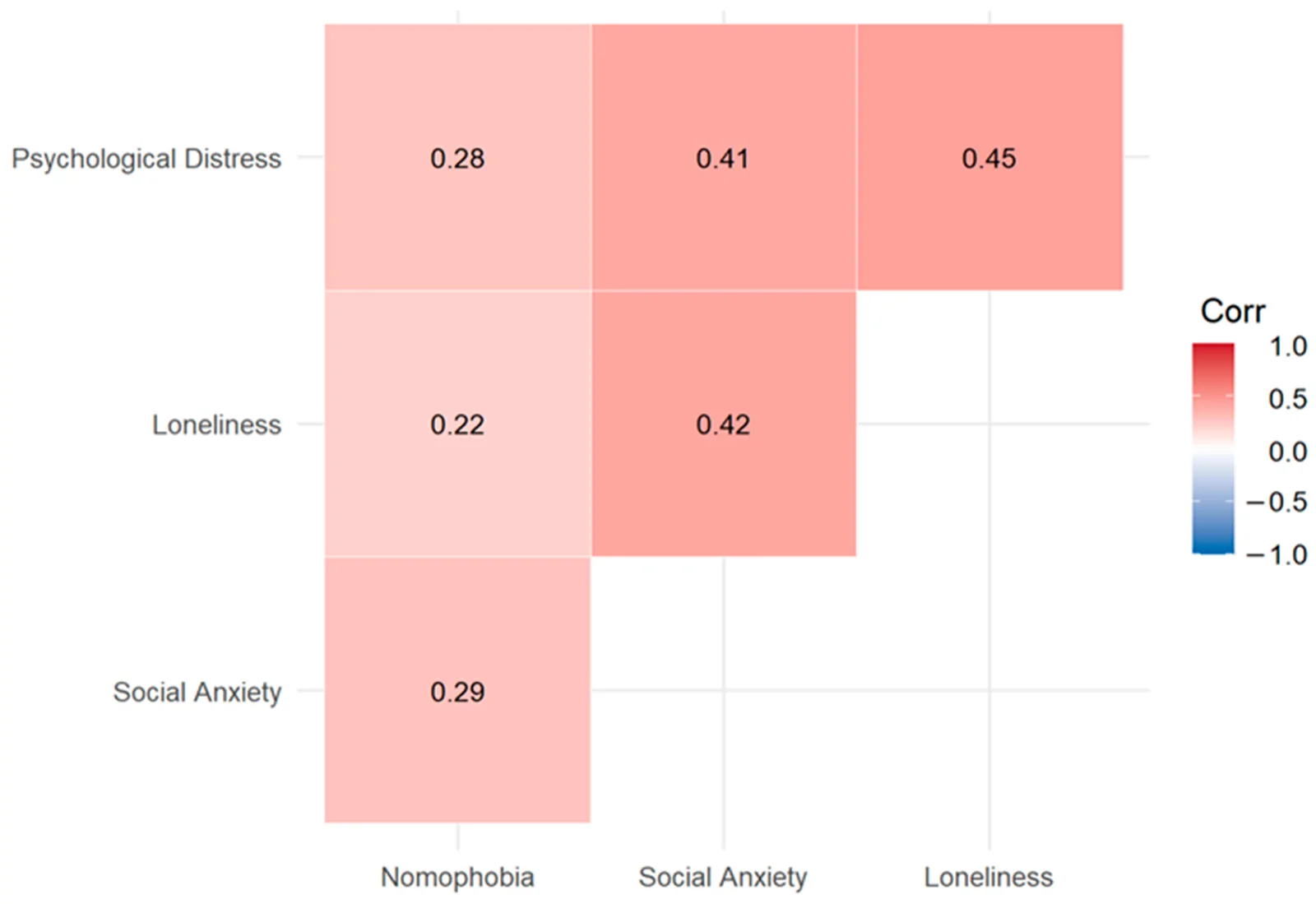
Correlation matrix of nomophobia, social anxiety, loneliness, and psychological distress.

Figure 1 presents correlation analysis between psychological constructs. The findings showed that nomophobia was positively correlated with social anxiety (*r* = 0.29) and psychological distress (*r* = 0.28), and a weak correlation with loneliness (*r* = 0.22). Social anxiety showed a moderate correlation with loneliness (*r* = 0.42) and psychological distress (*r* = 0.41). A higher correlation was found between loneliness and psychological distress (*r* = 0.45). This means that higher levels of loneliness were correlated with greater psychological distress. The correlations overall suggest that the constructs were related but conceptually distinct, thereby supporting their inclusion as separate latent variables in the subsequent structural equation model.

The integrated confirmatory factor analysis demonstrated an excellent fit to the data (χ^2^ = 326.57, df = 179, χ^2^/df = 1.82, CFI = 0.993, TLI = 0.992, RMSEA = 0.057, and SRMR = 0.053) (Table 2). This means that the hypothesized measurement model adequately represented the observed data. The abbreviated Nomophobia Questionnaire exhibited a two-factor structure, with standardized factor loadings ranging from 0.697 to 0.901 for the first factor and 0.772 to 0.891 for the second factor. Satisfactory standardized loadings were also found for the remaining constructs, ranging from 0.741 to 0.749 for Social Anxiety, 0.656 to 0.762 for Loneliness, and 0.666 to 0.744 for Psychological Distress. All standardized loadings exceeded the recommended threshold of 0.50 and were statistically significant (*p* < 0.001). This indicates that there was good indicator reliability and supported the construct validity of the measurement model. The Fornell–Larcker analysis supported adequate discriminant validity for all latent constructs (Supplementary Table S2). Specifically, the square root of the AVE for each construct exceeded its correlations with the remaining constructs. Although Social Anxiety and Loneliness were moderately correlated (r = 0.623), the square root of the AVE for Social Anxiety (√AVE = 0.746) and Loneliness (√AVE = 0.728) both exceeded their inter-construct correlation, supporting their empirical distinctiveness. Similar findings were observed for the remaining constructs, indicating satisfactory discriminant validity across the measurement model.

The findings of the structural equation model on the association between the two dimensions of nomophobia, social anxiety, loneliness, and psychological distress while adjusting for gender, smartphone use duration, and college affiliation were presented in Table 3. The model shown an excellent fit to the data (χ^2^ = 494.951, df = 233, CFI = 0.986, TLI = 0.988, RMSEA = 0.067, SRMR = 0.051). The fit supports the adequacy of the hypothesized structural model.

For social anxiety, the first nomophobia dimension, representing concerns related to access to information and communication, was positively associated with social anxiety (β = 0.228, *p* = 0.006). A positive association was found between gender (male) and social anxiety (β = 0.242, *p* = 0.005). In contrast, the second nomophobia dimension, reflecting continuous connectedness, smartphone use duration, and college affiliation, was not significantly associated with social anxiety.

For loneliness, social anxiety showed strong association (β = 0.561, *p* < 0.001). This means that students with higher levels of social anxiety were more likely to experience loneliness. We also found positive association between gender and loneliness (β = 0.314, *p* < 0.001). No significant association was found between smartphone use duration and college with loneliness.

With respect to psychological distress, loneliness was the only significant direct predictor (β = 0.479, *p* < 0.001). This suggests that higher levels of loneliness were associated with increased psychological distress. Although social anxiety (β = 0.181, *p* = 0.090) and the first nomophobia dimension (β = 0.195, *p* = 0.063) showed positive associations with psychological distress, the association did not reach statistical significance. The second nomophobia dimension, gender, smartphone use duration, and college affiliation were likewise not significantly associated with psychological distress.

Mediation analysis indicated that neither nomophobia dimension exerted a significant indirect effect on psychological distress through social anxiety alone. However, a significant sequential indirect effect was observed for the first nomophobia dimension through social anxiety and loneliness (β = 0.067, *p* = 0.042). This finding suggests that concerns related to access to information and communication contribute to increased psychological distress indirectly by increasing social anxiety, which subsequently increases feelings of loneliness. No significant sequential indirect effect was observed for the second nomophobia dimension.

In addition to the specific indirect effects, we examined the total indirect and total effects of the two nomophobia dimensions on psychological distress. The total indirect effect of Nomophobia Factor 1 (Information and Communication Access) was statistically significant (β = 0.111, 95% CI: 0.024–0.199, *p* = 0.013), indicating that its association with psychological distress was transmitted collectively through the proposed mediating pathways. The corresponding total effect was also significant (β = 0.324, 95% CI: 0.101–0.546, *p* = 0.004), demonstrating an overall positive association between this nomophobia dimension and psychological distress. In contrast, neither the total indirect effect (β = 0.065, 95% CI: −0.010 to 0.139, *p* = 0.089) nor the total effect (β = 0.063, 95% CI: −0.129 to 0.254, *p* = 0.522) of Nomophobia Factor 2 (Continuous Connectedness) reached statistical significance, suggesting that this dimension was not associated with psychological distress through the hypothesized mediating pathways.

## 4. Discussion

### 4.1. Principal Findings

This study examined the associations among nomophobia, social anxiety, loneliness, and psychological distress among university students in Riyadh, Saudi Arabia, using structural equation modeling. The findings showed that the nomophobia dimension reflecting concerns about losing access to information and communication was positively associated with social anxiety, whereas the dimension reflecting continuous connectedness was not independently associated with the study outcomes. Social anxiety was strongly associated with loneliness, and loneliness emerged as the only significant direct predictor of psychological distress. Furthermore, social anxiety and loneliness sequentially mediated the association between the information and communication dimension of nomophobia and psychological distress. The findings suggest that the association between nomophobia and psychological distress is primarily explained through interconnected psychological processes rather than a direct association. This means that social anxiety and loneliness are the main psychological pathways linking problematic smartphone dependence with poorer mental health among university students.

### 4.2. Comparison to Previous Studies

In our study, the nomophobia dimension related to concerns about losing access to information and communication was positively associated with social anxiety, whereas the dimension related to continuous connectedness did not demonstrate a statistically significant association. These findings suggest that different components of nomophobia may have distinct psychological implications and that anxiety arising from disrupted access to communication and essential information may be more strongly related to social anxiety than concerns about maintaining continuous online connectivity.

Our findings are consistent with a previous study reporting positive associations between nomophobia and social anxiety among university students and young adults [25]. Individuals with high social anxiety often perceive face-to-face interactions as stressful because of concerns about negative evaluation, embarrassment, or rejection [26]. Smartphones may therefore serve as a relatively safe and controllable means of communication, allowing socially anxious individuals to communicate while minimizing the uncertainty and emotional demands associated with direct interpersonal interactions [27].

Several psychological mechanisms may explain this association. Smartphones may function as a coping strategy for individuals with social anxiety by reducing exposure to anxiety-provoking social situations. Digital communication enables users to control the timing, content, and pace of conversations, thereby reducing perceived interpersonal risks. Repeated reliance on this form of communication may gradually reinforce avoidance of face-to-face interactions, strengthening both smartphone dependence and social anxiety [28]. Concerns about missing important information, messages, or opportunities for social interaction may heighten anticipatory anxiety, particularly among individuals who already experience heightened sensitivity to social evaluation. It is also possible that excessive dependence on smartphones may reduce opportunities to develop interpersonal communication skills, further perpetuating social avoidance and reinforcing anxiety in real-world social settings [29].

Interestingly, the second nomophobia dimension, related to continuous connectedness through notifications, social media, and online networks, was not significantly associated with social anxiety after adjustment for the other variables. This finding suggests that frequent engagement with digital platforms or concerns about remaining constantly connected may not necessarily reflect underlying social anxiety. Instead, these behaviors may be driven by habit, entertainment, information seeking, or social norms rather than fear of interpersonal evaluation. In contrast, concerns specifically related to losing access to communication and essential information appear to represent a more psychologically meaningful component of nomophobia in relation to social anxiety.

Another important finding of this study was the strong positive association between social anxiety and loneliness. This finding suggests that university students experiencing greater fear of social interactions and negative evaluation are more likely to report feelings of social isolation and lack of meaningful interpersonal relationships. The magnitude of this association highlights social anxiety as a key psychological correlate of loneliness within the proposed conceptual framework. This finding is consistent with previous research demonstrating that individuals with elevated social anxiety often experience difficulties initiating and maintaining satisfying interpersonal relationships [30,31,32]. Fear of embarrassment, rejection, or negative evaluation may lead socially anxious individuals to avoid face-to-face interactions, participate less frequently in social activities, and withdraw from opportunities to establish supportive social networks. Although such avoidance may temporarily reduce anxiety, it can inadvertently decrease the quantity and quality of social relationships, thereby increasing perceived loneliness. This mechanism is well supported by cognitive-behavioral models of social anxiety, which propose that avoidance behaviors maintain anxiety while simultaneously limiting opportunities to challenge maladaptive beliefs and develop positive social experiences [33].

Our findings concluded that loneliness was the only significant direct predictor of psychological distress in the structural model, whereas the direct associations of both nomophobia dimensions and social anxiety with psychological distress were no longer statistically significant after accounting for the proposed mediating pathways. Furthermore, social anxiety and loneliness sequentially mediated the association between the nomophobia dimension reflecting concerns about information and communication access and psychological distress. The strong association between loneliness and psychological distress is consistent with previous studies [15,34]. Persistent feelings of social isolation may reduce perceived social support, increase emotional vulnerability, and impair adaptive coping strategies, thereby increasing susceptibility to symptoms of anxiety and depression. The absence of a significant direct association between social anxiety and psychological distress after accounting for loneliness provides further insight into the psychological processes underlying the proposed model. Although social anxiety was associated with psychological distress in the expected direction, its association appeared to operate primarily through increased loneliness. This finding suggests that fear of social situations alone may not be sufficient to explain poorer mental health. Instead, the adverse psychological consequences of social anxiety may arise when persistent social avoidance limits opportunities to establish meaningful interpersonal relationships.

Our findings are also consistent with recent meta-analyses demonstrating that nomophobia is moderately associated with depression, anxiety, stress, and psychological distress across diverse populations [9,10]. Although these reviews consistently report significant overall correlations, most included studies relied on conventional correlation or regression analyses that did not explicitly evaluate mediating psychological processes. In contrast, structural equation modeling enabled us to simultaneously estimate direct and indirect associations while accounting for shared variance among the latent constructs. After social anxiety and loneliness were incorporated into the model, the direct association between nomophobia and psychological distress was attenuated and no longer statistically significant, whereas the sequential indirect pathway remained significant. This pattern suggests that the association between nomophobia and psychological distress may operate primarily through intermediary psychological mechanisms rather than through a direct relationship.

An important contribution of this study is the identification of a significant sequential indirect association between nomophobia and psychological distress through social anxiety and loneliness. This sequential pathway provides a more comprehensive understanding of the psychological processes through which problematic smartphone dependence may be associated with mental health outcomes than models examining only direct associations.

### 4.3. Strengths and Limitations

Our study has several strengths. It provides important insights into the psychological pathways linking nomophobia with psychological distress among university students. By simultaneously examining nomophobia, social anxiety, loneliness, and psychological distress within a single structural equation modeling framework, the study extends previous research that has largely focused on isolated associations between these constructs. The use of structural equation modeling enabled the simultaneous estimation of direct and indirect associations while accounting for measurement error, thereby providing a more comprehensive understanding of the relationships among the study variables. The inclusion of students from all three public universities in Riyadh further strengthens the study by increasing the diversity of the study population across academic disciplines and years of study. In addition, all psychological constructs were assessed using internationally recognized instruments that demonstrated satisfactory reliability, convergent validity, and excellent measurement model fit within the study population, supporting the robustness of the findings.

However, there are several limitations to acknowledge. The cross-sectional design represents an important limitation of this study. Although the hypothesized structural model was developed based on theoretical considerations and demonstrated excellent model fit, the cross-sectional design does not permit conclusions regarding temporal relationships or causality. Consequently, the observed direct and indirect pathways should be interpreted as statistical associations rather than causal mechanisms, and prospective longitudinal studies are needed to confirm the temporal ordering of the proposed relationships. Participation was voluntary, and despite implementing a university-wide recruitment strategy across all three public universities in Riyadh, self-selection bias cannot be completely excluded. It is possible that students with greater interest in smartphone use or mental health were more likely to participate, which may have influenced the observed estimates. The study was also limited to public university students in Riyadh, and caution is therefore warranted when generalizing the findings to students attending private universities, individuals outside the university setting, or populations from different cultural and educational contexts. An additional limitation is that the study evaluated an abbreviated two-factor version of the Nomophobia Questionnaire rather than the original 20-item, four-factor instrument. Although the abbreviated model demonstrated satisfactory psychometric properties within the present sample, its use may limit direct comparability with previous studies and meta-analyses based on the original instrument. Another limitation is that all variables were measured using self-administered questionnaires using QR codes. Although anonymous data collection and the use of well-validated instruments likely reduced reporting bias, self-reported measures remain susceptible to recall errors and social desirability bias. The relatively modest sample may have reduced the ability to detect smaller direct and indirect effects and may limit the generalizability of the findings. Furthermore, while the analyses adjusted for important demographic characteristics and smartphone use behaviors, other potentially influential factors, including personality traits, coping styles, perceived social support, academic stress, sleep quality, and pre-existing mental health conditions, were not assessed and may have contributed to the observed associations. Our study did not directly assess behavioral self-control or loss of control over smartphone use, such as the inability to regulate screen time or problematic smartphone use. Although the NMP-Q captures multiple dimensions of nomophobia, these related behavioral constructs may represent complementary mechanisms through which nomophobia contributes to psychological distress. Future longitudinal studies incorporating a broader range of psychosocial and behavioral factors, together with objective measures of smartphone use, would provide a more comprehensive understanding of the complex relationships among nomophobia, social anxiety, loneliness, and psychological distress.

## 5. Conclusions

This study showed that the association between nomophobia and psychological distress among university students is primarily explained through interconnected psychological processes rather than a direct relationship. Specifically, concerns related to losing access to information and communication were associated with greater psychological distress indirectly through increased social anxiety and loneliness. The findings of this study highlight the importance of addressing social anxiety and loneliness alongside problematic smartphone use when developing university mental health interventions. Future longitudinal studies are needed to establish the temporal relationships among these constructs and evaluate the effectiveness of interventions targeting these modifiable psychological pathways.

## Figures and Tables

**Table 1:** Sociodemographic characteristics, smartphone use patterns, and psychological measures of the study participants.

Characteristic	Category	*n* (%)/Mean ± SD
**Gender**	Male	140 (55.1)
	Female	114 (44.9)
**Age group (years)**	18 to 23	180 (70.9)
	24 years or older	74 (29.1)
**Year of study**	Years 1 to 2	50 (19.7)
	Years 3 to 4	188 (74.0)
	Year 5 or postgraduate	16 (6.3)
**College/Field of study**	Health Sciences	115 (45.3)
	Engineering/Science	35 (13.8)
	Education	35 (13.8)
	Business	41 (16.1)
	Humanities/Other	28 (11.0)
**Average daily smartphone use (hours)**	≤4 h	33 (13.0)
	4 to 8 h	166 (65.4)
	>8 h	55 (21.7)
Smartphone activity *	Social media	242 (95.3)
	Messaging and calls	172 (67.7)
	Entertainment	167 (65.7)
	Studying/Educational content	104 (40.9)
	News and browsing	67 (26.4)
	Gaming	62 (24.4)
**Psychological measures**	Nomophobia score	48.93 ± 12.94
	Social anxiety score	7.85 ± 2.40
	Loneliness score	5.80 ± 1.53
	Psychological distress (PHQ-4) score	8.82 ± 2.38

Abbreviations: PHQ-4, Patient Health Questionnaire-4; SD, standard deviation. * Participants could select more than one smartphone activity; therefore, percentages do not sum to 100%. **Note:** Data are presented as number (percentage) for categorical variables and mean ± standard deviation (SD) for continuous variables.

**Table 2:** Confirmatory factor analysis of the integrated measurement model.

Measurement Model	Result
**Model fit indices**	
**χ^2^ (df)**	326.57 (179)
**χ^2^/df**	1.82
**CFI**	0.993
**TLI**	0.992
**RMSEA**	0.057
**SRMR**	0.053
**Standardized factor loadings (λ)**	
**Nomophobia, (Information and Communication Access) (NM1 to NM6)**	0.697 to 0.901
**Nomophobia, (Continuous Connectedness) (NM7 to NM11)**	0.772 to 0.891
**Social Anxiety (SA1 to SA3)**	0.741 to 0.749
**Loneliness (LON1 to LON3)**	0.656 to 0.762
**Psychological Distress (MH1 to MH4)**	0.666 to 0.744

**Abbreviations:** χ^2^, chi-square statistic; df, degrees of freedom; CFI, Comparative Fit Index; TLI, Tucker–Lewis Index; RMSEA, Root Mean Square Error of Approximation; SRMR, Standardized Root Mean Square Residual.

**Table 3:** Standardized direct and indirect effects from the structural equation model.

Path	β	SE	95% CI	*p*-Value
**Direct effects**				
**Outcome: Social Anxiety**				
**Nomophobia, Factor 1 → Social Anxiety**	0.228	0.097	0.077 to 0.459	0.006
**Nomophobia, Factor 2 → Social Anxiety**	0.152	0.088	−0.018 to 0.329	0.079
**Gender → Social Anxiety**	0.242	0.146	0.123 to 0.696	0.005
**Smartphone use → Social Anxiety**	−0.036	0.060	−0.148 to 0.087	0.607
**College → Social Anxiety**	−0.099	0.045	−0.139 to 0.039	0.267
**Outcome: Loneliness**				
**Social Anxiety → Loneliness**	0.561	0.076	0.340 to 0.638	<0.001
**Nomophobia (Information and Communication Access) → Loneliness**	−0.037	0.109	−0.252 to 0.175	0.726
**Nomophobia (Continuous Connectedness) → Loneliness**	−0.030	0.093	−0.209 to 0.156	0.777
**Gender** (male) → **Loneliness**	0.314	0.136	0.197 to 0.729	<0.001
**Smartphone use → Loneliness**	−0.093	0.049	−0.166 to 0.028	0.162
**College → Loneliness**	−0.033	0.039	−0.091 to 0.061	0.705
**Outcome: Psychological Distress**				
**Loneliness → Psychological Distress**	0.479	0.149	0.216 to 0.800	<0.001
**Social Anxiety → Psychological Distress**	0.181	0.099	−0.026 to 0.361	0.090
**Nomophobia (Information and Communication Access) → Psychological Distress**	0.195	0.114	−0.011 to 0.436	0.063
**Nomophobia (Continuous Connectedness) → Psychological Distress**	−0.002	0.097	−0.193 to 0.189	0.983
**Gender → Psychological Distress**	−0.049	0.144	−0.359 to 0.207	0.599
**Smartphone use → Psychological Distress**	0.080	0.056	−0.047 to 0.173	0.260
**College → Psychological Distress**	0.120	0.044	−0.029 to 0.142	0.197
**Specific indirect effects**				
**Nomophobia (Information and Communication Access) → Social Anxiety → Psychological Distress**	0.045	0.031	−0.015 to 0.105	0.141
**Nomophobia (Continuous Connectedness) → Social Anxiety → Psychological Distress**	0.026	0.022	−0.017 to 0.069	0.239
**Nomophobia (Information and Communication Access) → Social Anxiety → Loneliness → Psychological Distress**	0.067	0.033	0.002 to 0.131	0.042
**Nomophobia (Continuous Connectedness)→ Social Anxiety → Loneliness → Psychological Distress**	0.039	0.024	−0.008 to 0.085	0.104
**Total indirect effects**				
Nomophobia (Information and Communication Access) → Psychological Distress	0.111	0.045	0.024 to 0.199	0.013
Nomophobia (Continuous Connectedness) → Psychological Distress	0.065	0.038	−0.010 to 0.139	0.089
**Total effects**				
Nomophobia (Information and Communication Access) → Psychological Distress	0.324	0.113	0.101 to 0.546	0.004

## Data Availability

The datasets used in this study are available from the first author on reasonable request due to participant privacy and ethical considerations.

## Supplementary Materials

The following supporting information can be downloaded at: https://www.mdpi.com/article/10.3390/healthcare14162441/s1, Table S1: Reliability and convergent validity indices for the nomophobia, social anxiety, loneliness, and psychological distress scales. Table S2. Fornell–Larcker criterion for assessment of discriminant validity.
