# Supplementary material for: Understanding the Relationship Between Nomophobia and Psychological Distress Among University Students: The Sequential Mediating Roles of Social Anxiety and Loneliness
Source: Healthcare (Basel). 2026 Aug 7;14(16):2441. doi: 10.3390/healthcare14162441 (PMC13512280; doi:10.3390/healthcare14162441)
Supplement: Supplementary file 1 [file healthcare-14-02441-s001.zip › healthcare-4448560-supplementary.pdf]

Supplementary results

Understanding the Relationship Between Nomophobia and Psychological Distress Among University Students: The Sequential Mediating Roles of Social Anxiety and Loneliness

Bandar S. Alharbi[1*], Majed M. Aljabri[1]

[1]Community and Psychiatric Mental Health Nursing Department, College of Nursing, King Saud University, Riyadh 12375, Saudi Arabia

[*] Corresponding author (Email: banalharbi@ksu.edu.sa)

Table S1: Reliability and convergent validity indices for the nomophobia, social anxiety, loneliness, and psychological distress scales.

**Scale**	**Ordinal α**	**ω**	**CR**	**AVE**
Nomophobia Information	0.902	0.901	0.901	0.638
Nomophobia Communication	0.918	0.909	0.909	0.703
Social Anxiety	0.787	0.748	0.748	0.556
Loneliness	0.752	0.688	0.688	0.530
Psychological Distress	0.804	0.754	0.754	0.510

Table S2. Fornell–Larcker criterion for assessment of discriminant validity

**Construct**	**Nmph_1**	**Nmph_2**	**Social Anxiety**	**Loneliness**	**Psychological Distress**
Nomophobia 1	**0.799**				
Nomophobia 2	0.775	**0.838**			

Social Anxiety	0.385	0.395	**0.746**		
Loneliness	0.215	0.274	0.623	**0.728**	
Psychological Distress	0.353	0.332	0.532	0.597	**0.714**
